# Editorial: Control of Adipocyte Differentiation and Metabolism

**DOI:** 10.3389/fendo.2015.00132

**Published:** 2015-08-28

**Authors:** Encarnación Capilla, Isabel Navarro

**Affiliations:** ^1^Department of Physiology and Immunology, University of Barcelona, Barcelona, Spain

**Keywords:** adipocytes, adipokines, adipogenesis, obesity, insulin resistance, brown fat, thermogenesis

For many years, adipose tissue was considered a passive tissue with the only function of storing excess energy as triglycerides and releasing fatty acids according to necessity. Now it becomes increasingly clear that adipose tissue is an endocrine organ that plays a critical role in modulating energy expenditure and homeostasis. White adipose tissue is thus responsible for the secretion of an array of signaling molecules, termed adipokines that function as classic hormones in other target tissues and whose secretion is affected by metabolic dysregulation (1). Furthermore, adipose tissue has the capacity to grow not only by hyperplasia through formation of new adipocytes from precursor cells but also by hypertrophy of existent adipocytes (2), which is essential for the correct function of the cell and metabolic control, and can be altered in different pathologies, such as obesity.

This Research Topic contains four review articles. Pessin and Kwon (3) examine the recent progress regarding the physiological and molecular functions of adipokines in obesity-induced inflammation and insulin resistance. The molecular mechanisms of obesity-associated type 2 diabetes are still unclear; however, recent studies have shown that low-grade chronic inflammation is an important factor in its pathogenesis in humans and rodent animal models (4). It is considered that obesity-induced insulin resistance results, at least in part, from an imbalance in the secretion of pro- and anti-inflammatory adipokines, which are reviewed in this manuscript. It is becoming clear that targeting inflammatory signaling pathways can be an effective approach in the treatment of insulin resistance.

Insulin resistance, inflammation, and cardiovascular diseases are associated with abdominal obesity. Thus, fat distribution (proportions of visceral and subcutaneous fat) and tissue composition can be metabolic health determinants (5). In this sense, the review of Ma et al. (6) analyzes the transcriptional regulation of adipose tissue differentiation and expansion into different depots of white and brown fat. Modulation of the function of these different adipose tissue depots opens novel avenues in obesity treatment. One exciting prospect in this area is to increase the amount and/or activity of brown adipocytes, which dissipate energy in contrast with white adipocytes. Recent discoveries of metabolically active brown adipose tissue in adult humans (7, 8) made it a potentially attractive target for development of anti-obesity therapeutics. Dempersmier and Sul (9) highlight the recent advances in the understanding of brown and beige/brite adipocytes focusing in the transcriptional regulation for development and function with the aim of finding new therapeutic targets. This revision reveals the critical transcriptional factors that have been identified as specific regulators of preadipocyte determination and differentiation of both white and brown adipocytes.

Control of brown adipocyte differentiation has an increasing interest as reflected in the review of the research topic by Hudak and Sul (10) that analyzed in detail the role of Pref-1 as a negative regulator of adipogenesis, which also prevents brown adipocyte differentiation and its thermogenic function. The signaling pathway of Pref-1 and the regulation of its expression by different factors are highlighted in this review indicating the basic and applied interest of these transcriptional factors controlling adipogenesis.

Overall, the review articles in this research topic have covered new insights on the control of different adipose tissue types’ differentiation, metabolism, and adipokines production and function in relation to different pathophysiological states offering new ideas for future investigations for therapeutic interventions.

## Conflict of Interest Statement

The authors declare that the research was conducted in the absence of any commercial or financial relationships that could be construed as a potential conflict of interest.
